# Observations upon the Phenomena and Treatment of Tetanus, with Cases

**Published:** 1809-06

**Authors:** John Howship


### *Observations upon the Phenomena and Treatment of Tetanus, with Cases.* By John Howship.

### *A Case of Tetanus, shewing the Inefficacy of Mercury.*

A Middle aged man, of the name of Robinson, boatswain on board H. M. S. Bellerophon, was wounded by a grape shot, that passed through the hand, producing a complicated laceration of the soft parts, with fracture of the bones. This was unattended with any peculiar circumstances earlier than the ninth day, at which time he mentioned that he experienced some difficulty in swallowing. He felt no inconvenience confined particularly to the jaws, but spoke of the impediment in deglutition as his only complaint. He was thirsty, and frequently desired to drink; but whenever he made the effort to swallow, the attendants say he made an odd sort of noise in the the throat, but did not succeed in the attempt. He suffered comparatively very little pain, except when he provoked the spasm by his frequent exertion in swallowing. From the muscles concerned in deglutition, the affection soon spread, according to its usual course, the jaws being fixed together, and the muscles about the neck and back becoming rigid and stiff, subject, together with the muscles of respiration and those of the extremities, to frequent and violent fits of spasmodic contraction. The complaint continued to increase until, upon the eleventh day, blisters were applied to the neck, and mercury both externally and internally exhibited. On the twelfth day he died. It is to be observed, that some time before the coming on of the fit which terminated fatally, this person was evidently under the influence of the mercurial excitement. The gums were rendered very full and fœtid, although the salivation was only in its commencement.

*A Case that proves the Irritation bringing on Tetanus is not peculiar to lacerated or to punctured wounds, sometimes arising after the fairest Incision.*

W. Meath, a stout healthy man, thirty years of age, had his leg shot away during an engagement, after the termination of which, the limb was removed by amputation above the knee. This man went on well till the ninth day, when the symptoms of lock jaw came on. Upon the day following mercury was given both by external and internal application. This man, however, was lost, although he was in a state of perfect and complete salivation thirty-six hours before he died.

This was one of the cases, in which Mr. Gardner, the senior Surgeon of the Navy Hospital at Gibraltar, gave a trial to the effects of pressure, under the persuasion that it might alleviate the severity of the spasms.

A screw tourniquet was applied high up on the thigh, the compress being deposited in the manner usual when it is intended to suspend the circulation in the limb. Upon the instrument being brought into action, it was observed that a moderate and even a considerable extent of pressure, produced no apparent variation whatever in the appearances of the complaint. The spasms were neither less frequent nor less violent than they were before the ligature was tightened; when, however, the screw was turned to the greatest extent, or as far as the yielding structure of the soft parts would allow, there was certainly an effect produced; the man said that he found the violence of the pain diminished, and therefore, although the contractions were not less frequent, his sufferings from them were in some degree relieved. He expressed a strong desire that the instrument should be allowed to remain, and was disappointed upon its being soon afterwards removed.

*Case of Tetanus, in which the Irritation through the wounded Nerve manifested itself from both Extremities of the Nervous Trunk.*

J. Cairns, a thin young man, seaman on board his Majesty's ship Colossus, was wounded by a musquet shot in the left groin; the ball passed out behind the hip on the same side. The man said that his wound never gave him much pain till the eighth day, when he experienced a terrible and new sort of pain catching him about the groin and hip, just as if something was snatching and wringing off the thigh. Upon careful inquiry it was ascertained, that the pain always commenced at the wounded part, passing from thence downwards, and "out at the toe ends."

On the ninth day he complained of not being able to open his jaws so freely as usual; but in this part he experienced no distress, nor felt any pain; he merely found difficulty in separating the jaws. His complaints were on the tenth day altogether worse; he frequently at this time felt severe pain about the jaws; at the same time remarking, with apparent surprise, that he now felt no pain in or about the wound. The pulse was in this case regular, soft, and equal.

With regard to his treatment, blisters were, on the 9th day, laid upon the sides of the throat, but to no purpose. The tenth was occupied in exhibiting mercury by friction, and pills. On the 12th he spit more than usual, but the following night he expired; although, according to the report of those who lay near him, he did not seem to suffer very severely, nor complain of particular pain in any part. On the contrary, he may be said to have breathed his last in a state of tranquillity unusual in this complaint. —Towards the last hours of this poor man's sufferings the spasms, though less violent than in many other instances, yet were very frequent; for the last two days he had rarely more than five minutes interval.

*Scarborough, May* 10, 1809.

(To be Continued.)

Dr. Alder's

